# Flavonoid Composition and Antioxidant Activity of *Tragia volubilis* L. Methanolic Extract

**DOI:** 10.3390/plants12173139

**Published:** 2023-08-31

**Authors:** Juan Carlos Romero-Benavides, Nora Cecilia Atiencie-Valarezo, Rodrigo Duarte-Casar

**Affiliations:** 1 Departamento de Química, Facultad de Ciencias Exactas y Naturales, Universidad Técnica Particular de Loja, Loja 110108, Ecuador; ncatiencie@utpl.edu.ec; 2 Maestría en Química Aplicada, Facultad de Ciencias Exactas y Naturales, Universidad Técnica Particular de Loja, Loja 110108, Ecuador; rduarte@utpl.edu.ec

**Keywords:** *Tragia volubilis* L., *Euphorbiaceae*, Ecuadorian medicinal plants, phytochemicals, flavonoids

## Abstract

Several species from the genus *Tragia* L. in the family *Euphorbiaceae* are part of the ethnomedicine of traditional cultures, and have a variety of uses. *Tragia volubilis* L. is a species spread through tropical America and Africa with several ethnomedical uses, particularly for wound healing and reproductive issues. In this study, we assess the phytochemical composition and antioxidant activity of the methanolic extract of the aerial parts of *T. volubilis* collected in southern Ecuador. The phytochemical screening of the extract shows the preliminary presence of carbohydrates, alkaloids, flavonoids, and tannins. The extract shows an Antioxidant Activity Index of 1.14, interpreted as strong antioxidant activity. Four flavonoid compounds were isolated through chromatographic procedures and identified through NMR spectroscopy: avicularin, quercitrin, afzelin, and amentoflavone. The biological activity of these compounds matches the ethnopharmacological uses of the species. This is the first phytochemical study of *T. volubilis* and supports its traditional medicinal uses.

## 1. Introduction

Utilizing plants for medicinal purposes is prevalent among several animal species; great apes and humans are but examples of a widespread practice [1]. Humanity has harnessed the health-enhancing properties of plants since prehistoric times. Early evidence shows the use of medicinal plants by our Neanderthal ancestors [2]. Even today, about 80% of the world’s population relies on herbal medicines to preserve and promote their well-being [3].

Ethnopharmacology is an interdisciplinary field that studies the biologically active agents recognized and used by man and the cultural heritage that surrounds their use. It is an important drug discovery approach. Traditional wisdom has converged with modern research methods and tools, resulting in the validation of many ethnopharmacological claims and the development of new and improved drugs and treatments from traditional medicinal plants [4].

### The Genus Tragia (Euphorbiaceae)

*Euphorbiaceae* is a plant family abundant in medicinal species. This abundance is attributed to the vast geographical distribution and, thus, the variety of environmental stressors that affect the species of the family, to which they react through the production of a variety of secondary metabolites [5]. Within this family, the *Tragia* Plum ex L. genus, present worldwide in the intertropical region, is traditionally used in African and Asian traditional medicinal systems, i.e., Ayurveda and Siddha [6], for a wide spectrum of ailments [7], as food [8], and for other uses, such as a low-toxicity irritant, evaluated as a riot control agent [9]. The species native to America are less studied, and, while traditionally used, do not seem to be part of documented medicinal systems. Around 26 of the more than 150 species in the genus are reported as medicinal, mostly as treatments for conditions of the genitourinary, nervous, and digestive systems, infections, and cancer. Most research to date focuses on four Asian and African species: *Tragia involucrata* Linn., *Tragia spathulata* Benth., *Tragia benthamii* Baker, and *Tragia plukenetii* Radcl. Research on the genus centers on antidiabetic and anti-inflammatory activity. Additionally, investigations have revealed significant biological activity and low toxicity in relation to its antimalarial, antibacterial, antitumor, and diuretic effects [10].

More than sixty New World *Tragia* species comprise almost 40% of the total species in the genus. Despite this abundance, less research has been conducted on the New World than on the Old World *Tragia* species, even though there is evidence of its use by First Nations [11] before the Hispanic contact [12]. The eight New World medicinal *Tragia* species mentioned in the literature and their uses are shown in Table 1, along with their uses.

In addition to medical uses, there are other reported activities of the New World species: *T. biflora* is allelopathic against water plants; pounded, boiled *T. brevispica* taken orally is reportedly aphrodisiac in Uganda [20]; and *T. gracilis* is used in Cuba for *Santería* religious uses [21].

**Figure 1:**
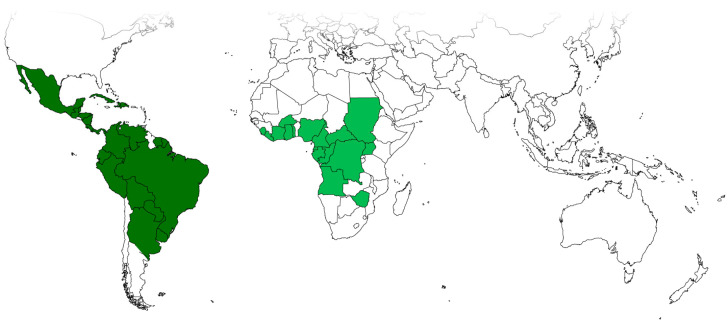
Geographical distribution of *T. volubilis*. Green: native. Light green: possibly introduced. Source [25].

Among the American species, the most widely distributed, and the lectotype chosen by Linnaeus for the genus [22], is *T. volubilis*, present not only in intertropical America and the Caribbean but also in West and Central Africa, where it is presumably an introduced species [23,24]. Its distribution is shown in Figure 1, encompassing the seasonally dry tropical biome from southern USA to Central Argentina in America and from Sudan to Zimbabwe in Africa.

The species is called “twining Tragia” in Jamaica due to its growth habit, and *Pringamoza morada* in Cuba due to its purple flowers [26]. Most of its vernacular names, such as “fireman” or “cowitch,” stem from the intense irritation it produces, due to its stinging hairs (raphides) tipped with calcium oxalate crystals that cause a painful, transient contact dermatitis that disappears, leaving no trace [27]. On contact irritation tests, the intensity of its sting is one out of three, equivalent to low intensity [28].

**Figure 2:**
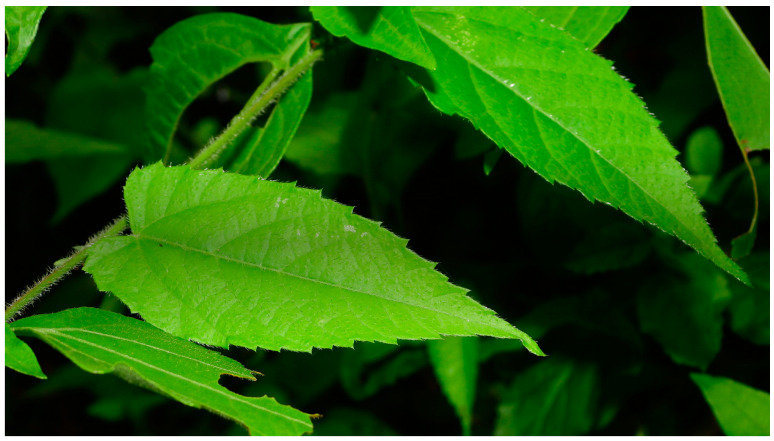
*Tragia volubilis* plant, showing serrated leaves and stinging hairs. Photo by Sebastien Sant, CC-BY-NC-4.0. French Guiana, France.

*T. volubilis* is a perennial, climbing herb or subshrub, either totally voluble or with voluble apexes, with simple, alternate, usually serrated leaves and simple, urticant hair pubescences. Paniculoid inflorescences are positioned terminally and opposite the leaves with 1- ∞ pistillate flowers. They consist of a main terminal axis with 3- ∞ staminate flowers and persistent bracts [29]. Figure 2 shows the serrated leaves and stinging hairs of the plant.

The species is part of the Maya tradition in Mesoamerica, where it is called “Quetzalcoatl’s herb,” where Quetzalcoatl or Kukulkan—the feathered serpent—is the supreme Maya deity. The plant twines like a serpent and is covered in “feathers”—actually raphides—to resemble the supernatural being [30]. In the sixteenth-century “History of the Plants of New Spain” by Francisco Hernández, it is said that its leaves “cure madness when drunk” [12]. The species is considered urticant, rubefacient, and medicinal. Its published ethnomedical uses are listed in Table 2.

*T. volubilis* is an insufficiently studied species. Most research on the species, searched through Dimensions [40] using “Tragia volubilis” on a full-text search, shows the species is mentioned in 168 publications, with ecology (78), plant biology (23), and both agriculture and environmental science (19) as major disciplines. There are no health sciences or chemical studies reported for the species directly. There is as yet no phytochemical study on this species, and there is no verification of its reported medicinal properties beyond its confirmed use as a diuretic [41,42].

The objective of this work is to provide a first phytochemical study of the species and to suggest preliminary molecular grounds for the reported ethnopharmacological uses.

## 2. Results and Discussion

The results of this phytochemical study of *T. volubilis* are as follows.

### 2.1. Extract

In total, 1987 g of fresh plant material yielded 1287 g of dried aerial parts of *T. volubilis*, from which 62.87 g of methanolic extract was obtained (yield: 7.39%). The yield is similar to those of methanolic extracts of other *Euphorbiaceae* species (4.11–8.85%) [43], and also comparable to *T. involucrata* leaf extract at 6.75% [44]. Most methanolic *Tragia* extracts reported are root extracts, mainly those of *T. involucrata* [45].

### 2.2. Phytochemical Screening

The methanolic extract of aerial parts of *T. volubilis* was subject to a preliminary phytochemical screening to show the compound classes present (Table 3). Terpenoids showed a scant presence; flavonoids and carbohydrates exhibited a moderate presence; and alkaloids and tannins showed a strong presence. These compound families are similar to those found in other *Tragia* species, such as *Tragia involucrata* L. [46,47], *Tragia pungens* (Forssk.) Müll. Arg. [48], and *Tragia benthamii* Baker [49].

Terpenoids, alkaloids, and flavonoids exhibit biological activity consistent with the ethnopharmacological uses reported for the species, notably antimicrobial action that could underlie wound healing, anti-STD, and anti-ulcer activity [50]. Flavonoids [51] and terpenoids [52] also exhibit blood-pressure-lowering effects. Flavonoids and tannins are phenolic compounds with well-known antioxidant biological activity, which could contribute to the reported anti-cancer properties.

### 2.3. Antioxidant Activity

The extract exhibits strong antioxidant activity measured through the Antioxidant Activity Index (AAI > 1). The antioxidant activity analysis is shown in Table 4.

The high antioxidant capacity of the extract can be ascribed to its phenolic content, similar to the polar extracts of other species (*T. involucrata*) [53].

**Table 4:** Antioxidant activity of *T. volubilis* methanolic extract.

TPC mg GAE/ g Extract	ABTS μmol TE/ g Extract	FRAP μmol TE/ g Extract	DPPH μmol TE/ g Extract	IC_50_ mg Extract/ mg DPPH	AAI [DPPH] (μg mL^−1^)/IC_50_
127 ± 2	2004 ± 36	1250 ± 15	585 ± 5	1.30 ± 0.06	1.14 ± 0.01

All values are average ± SD of three repetitions. TPC: Total Phenolic Content. GAE: Gallic acid equivalent. TE: Trolox (6-hydroxy-2,5,7,8-tetramethylchroman-2-carboxylic acid) equivalent. ABTS: 2,2′-azino-*bis* (3-ethylbenzothiazoline-6-sulfonic acid) method. FRAP: Ferric reducing antioxidant power. DPPH: 2,2-diphenyl-1-picrylhydrazyl method. IC_50_: Half maximal inhibitory concentration. AAI: Antioxidant Activity Index [54].

### 2.4. Compounds

**Figure 3:**
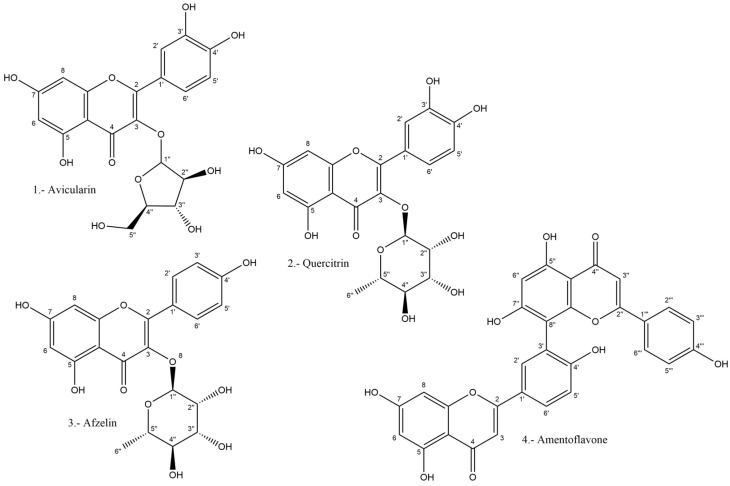
Compounds isolated from *T. volubilis* methanolic extract.

From the dechlorophyllated methanolic extract, four compounds, (**1**) avicularin, (**2**) quercitrin, (**3**) afzelin, and (**4**) amentoflavone, were isolated through chromatographic techniques and identified through NMR spectroscopy. The structures are shown in Figure 3.

Avicularin was isolated by preparative TLC on direct silica F_254_ using 120 mL EtOAc:HOAc:H_2_O 18:1:1 (2.1 mg). Its R_F_ was 0.65. The compound is a quercetin 3-*O* glycoside, where the sugar moiety is an arabinose unit. It was first isolated from the common knotgrass (*Polygonum aviculare*) and is considered to contribute to the hypoglycemic activity of *Psidium guajava* [55] and to the antibacterial and antifungal activity of *Hypericum perforatum* [56].

Quercitrin was isolated by preparative TLC on direct silica F_254_ using 120 mL DCM:MeOH 90:10 and two drops of water (5.6 mg). Its R_F_ was 0.42. The compound is a quercetin 3-*O* glycoside, with rhamnose—a deoxy sugar—unit as the sugar moiety. It was isolated from oak (*Quercus* spp.), hence its name. It is used as a yellow dye and also as a bioactive product with varied biological effects [57].

Afzelin was isolated by preparative TLC on reverse-phase silica using MeOH:H_2_O 65:35 (3.3 mg). Its R_F_ was 0.5. The compound is a kaempferol 3-*O* rhamnoside. It was first isolated from the fragrant water lily (*Nymphaea odorata*) [58]. It shows antibacterial and antitumor activity.

Amentoflavone was isolated as a yellow solid on Sephadex LH-20 on a microcolumn using MeOH:H_2_O 60:40 as eluent (2.2 mg). Its R_F_ was 0.34. The compound is a biflavonoid: 3′-8″ apigenin, originally isolated from *Ginkgo biloba.* It is considered a multifunctional compound [59].

The ^1^H and ^13^C NMR spectra information of the identified compounds follows.

**Avicularin (1)**: ^1^ H–NMR (500 MHz, CD_3_OD, δ ppm, *J* in Hertz): 3.50 (2H, m, H-5″), 3.86 (1H, m, H-4″), 3.91 (1H, dd, 5.4; 3.0, H-3″), 4.33 (1H, dd, 1.1; 3.0, H-2″), 5.47 (1H, d, 1.1, H-1″), 6.21 (1H, d, 2.1, H6), 6.40 (1H, d, 2.1, H-5′), 6.40 (1H, d, 2.1, H-8), 7.49 (1H, d, 8.4, H-6′), 7.53 (1H, d, 2.1, H-2′).

^13^C NMR (125 MHz, CD_3_OD, δ ppm, Carbon number): 61.3 (5″), 77.3 (3″), 82.0 (2″), 86.6 (4″), 93.4 (8), 98.6 (6), 104.3 (10), 108.2 (1″), 115.1 (2′), 115.6 (5′), 121.6 (1′), 121.7 (6′), 133.5 (3), 145.0 (3′), 148.9 (4′), 157.6 (9), 159.1 (2), 162.6 (5), 165.4 (7), 177.3 (4).

**Quercitrin (2)**: ^1^H–NMR (500 MHz, CD_3_OD, δ ppm, *J* in Hertz): 0.93 (3H, d, 6.2, H-6″), 3.33 (1H, m, H-4″), 3.41 (1H, m, H-5″), 3.74 (1H, dd, 9.5 3.3, H-3″), 4.21 (1H, m, H-2″), 5.30 (1H, d, 1.1 H-1″), 6.19 (1H, d, 1.5, H-6), 6.35 (1H, brs, H-8), 6.90 (1H, d, 8.3, H-5′), 7.30 (1H, dd, 8.4 1.9, H-6′), 7.33 (1H, d, 1.8. H-2′).

^13^C NMR (125 MHz, CD_3_OD, δ ppm): 16.2 (6″), 70.5 (2″), 70.6 (3″), 70.7 (5″), 71.8 (4″), 93.4 (8), 98.5 (6), 104.5 (1″), 104.5 (10), 115.0 (5′), 115.5 (2′), 121.5 (6′), 121.6 (1′), 134.8 (3), 145.0 (3′), 148.5 (4′), 157.1 (9), 157.9 (2), 161.7 (5), 164.5 (7), 178.3 (4).

**Afzelin (3)**: ^1^ H–NMR (500 MHz, Acetone D6, δ ppm, *J* in Hertz):

0.89 (3H, d 6, H-6″), 3.3 (1H, m, H-4″), 3.32 (1H, m, H-5″), 3.67 (1H, m, H-3″), 4.21 (1H, m, H-2″), 5.54 (1H, d 1.5, H-1″), 6.26 (1H, d 2.0, H-6), 6.47 (1H, d 2.0, H-8), 7.01 (1H, d 9, H-3″), 7.02 (1H, d 9, H-5′), 7.84 (1H, d 8.5, H-6′), 7.86 (1H, d 8.5, H-2′), 9.71 (1H, s, 5-OH).

^13^C NMR (Acetone D6, δ ppm): 16.9 (6″), 70.4 (5″), 70.6 (2″), 71.2 (3″), 72.1 (4″), 93.6 (8), 98.6 (6), 101.8 (1″), 104.5 (10), 115.4 (3′), 115.4 (5′), 121.7 (1′), 130.8 (2′), 130.8 (6′), 134.8 (3), 157.1 (4′), 157.5 (9), 159.9 (2), 162.4 (5), 164.2 (7).

**Amentoflavone (4)**: ^1^ H–NMR (500 MHz, CD_3_OD, δ ppm, *J* in Hertz):

6.20 (1H, d, 2.4, H-6), 6.32 (1H, s, H-6″), 6.43 (1H, d, 2.4, 8), 6.60 (1H, s, H-3), 6.68 (1H, s, H-3″), 6.76 (1H, d, 8.2, H-3′′′), 6.76 (1H, d, 8.2, H-5′′′), 7.12 (1H, d, 8.2, H-5′), 7.70 (1H, d, 8.2, H-2′′′), 7.70 (1H, d, 8.2, H-6′′′), 7.94 (1H, dd, 2.2 8.4, H-6′), 8.22 (1H, d, 2.2, H-2′),

^13^C NMR (CD_3_OD, δ ppm): 94.9 (8), 99.9 (6), 100.9 (6″), 103.4 (3), 103.4 (3″), 104.3 (8″), 105.0 (10), 105.6 (10″), 116.3 (3′′′), 116.3 (5′′′), 118.5 (5′), 121.5 (3′), 123.3 (1′′′), 123.4 (1′), 127.5 (6′), 128.7 (2′′′), 128.7 (6′′′), 132.2 (2′), 159.0 (7), 159.0 (9), 159.0 (9″), 161.8 (4′′′), 162.1 (7″), 162.4 (2), 162.4 (4′), 164.4 (2″), 164.5 (5), 165.6 (5″), 183.0 (4″), 183.1 (4).

All the identified compounds exhibit potent antioxidant activity, which is summarized in Table 5.

The structure of the isolated compounds supports the antioxidant activity: they are all B-ring hydroxylated compounds, which is the most significant indicator of ROS and RNS scavenging activity, and the vicinal double OH groups in the B-ring of compounds **1** and **2** indicate strong lipid peroxidation inhibition [63]. Rhamnosides, though, present less metal-chelating activity than 6″OH glycosides [64].

Structure-activity relationships for cellular antioxidant effects are 3′,4′ *o* dihydroxyl group, 2,3 double bond conjugated with 4-keto group, and 3-hydroxyl group. Compounds **1** and **2** satisfy all the conditions.

Flavonols, such as compounds **1**, **2**, and **3**, are among the most potent antibacterial flavonoids through mechanisms including interference with fatty acid elongation and are capable of synergistically reducing antibiotic resistance. Flavones such as **4** inhibit bacterial growth by forming complexes with cell wall components [65].

The isolated flavonoids show promising biological activity, which is exemplified in Table 6. There is a good overlap between the ethnopharmacological uses of *T. volubilis* and the biological activity of the identified compounds.

Compounds **2** and **3** exhibit biological effects in methanolic extracts due to their antioxidant capacity [70]. Compound **3** has undergone pre-clinical studies against lung cancer, also due to its antioxidant activity [75]. The antioxidant activity of *Tragia* spp. extracts underlie several ethnopharmacological uses which have been validated in vivo and have undergone clinical trials [76,77,78].

Extracts from the leaves of other *Tragia* species also contain potent antioxidant flavonoids, which are quercetin and kaempferol glycosides, for example, *T. plukenetii* [76] and *T. involucrata* [79]. Quercetin and kaempferol—which are metabolic products of their glycosides—are among the most frequently studied flavonoids and are recommended as dietary supplements due to their high biological activity [80].

## 3. Materials and Methods

### 3.1. Plant Material

**Figure 4:**
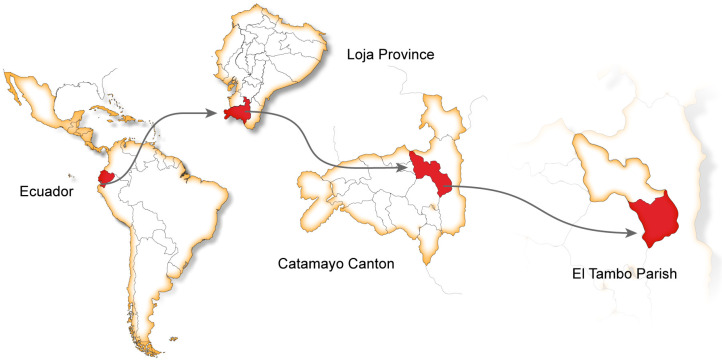
Collection area of *T. volubilis* L. used in this study.

Aerial parts of *Tragia volubilis* L. were collected in El Tambo, -Catamayo, Loja province in southern Ecuador. Coordinates: 04°07′13.3″ S; 79°18′11.9″ W; 1600 m ASL (Figure 4). Species identification was performed by Fani Tinitana, PhDPh.D., and a voucher specimen was deposited at the Herbarium of Universidad Técnica Particular de Loja, Ecuador. The specimen was collected in compliance with the Framework Contract MAE-DNB-CM-2016-0048 dated 20 September 2016. The plant material was dried for 7 days at 30 °C under airflow.

### 3.2. Preparation of the Extract

Because it is common for methanolic extracts to show higher biological activity than aqueous extracts [81], and the fact that methanol is the primary solvent used to date in *Tragia* species studies, with 47% of the studied *Tragia* spp. extracts [10], it was decideda decision was made to focus the present study on the methanolic extract rather than the aqueous extract, even though most ethnomedical uses employ aqueous extracts and decoctions. The dry plant material was extracted by static maceration for 3 days with analytical-grade methanol purchased from Merck, then filtered, and concentrated on a rotary evaporator (Buchi R210, Flawil, Switzerland) to yield the *Tragia volubilis* methanolic extract, which was stored at −18 °C.

### 3.3. Phytochemical Screening

The phytochemical screening of the extract was performed according to the methodology of Mandal et al. [82]. The Biuret copper -complex formation test was used for the detection of proteins; positive controls used were powdered milk, egg albumin, and glutamic acid. The Fehling test for reducing sugars was used for the detection of carbohydrates; positive controls were sucrose and glucose. Sudan fat-soluble dye was used for screening lipids; the positive control used was vegetable oil. The Dragendorff potassium tetraiodobismuthate test was used for alkaloids; the positive control was caffeine. The Lieberman Burchard acetic anhidride anhydride test was used for terpenoids; the: positive control was Argentatin B. The Shinoda magnesium and hydrochloric acid test was used for flavonoids; the positive control was hesperidin. The foam test was used for saponins; the: posivitepositive control was grated raw potato. The Bornträger test was used for quinones; the positive control was hydroquinone. The ferric chloride assay was used to test for phenolics-–tannins, with vanillin used as the positive control.

### 3.4. Antioxidant Activity

Total phenolic content was determined through the Folin-–Ciocâlteu method [83]. To a diluted sample of the extract, Folin-–Ciocâlteu reagent was added, and the wells were homogenized for 10 min. A total of 7.5% *w*/*w* Na_2_CO_3_ solution was added, and the wells were homogenized again for 5 min. Absorbance was measured at 760 nm on a Bio Tek Epoch 2 microplate reader (Winooski, VT, USA), and values were compared to a gallic acid calibration curve. Results are expressed in gallic acid equivalents (GAE) per gram of extract.

Antioxidant capacity was measured through the following tests: ABTS (2,2′-azino-bis (3-ethylbenzothiazoline-6-sulfonic acid)) [84], FRAP (Ferric reducing antioxidant power) [85], and DPPH (2,2-diphenyl-1-picrylhydrazyl) methods [86]. All antioxidant activity was determined against a Trolox (6-hydroxy-2,5,7,8-tetramethylchroman-2-carboxylic acid) standard. Antioxidant Activity Index (AAI) was calculated as the quotient between the final DPPH concentration and the IC_50_, providing a value that is independent of both the nature of the sample and DPPH concentrations [54]. AAI relates to antioxidant activity in plant extracts as follows: AAI < 0.5 is considered poor antioxidant activity. An AAI between 0.5 and 1.0 is considered moderate antioxidant activity. Values between 1.0 and 2.0 are considered high antioxidant activity, and an AAI > 2.0 is considered very high antioxidant activity. All tests were repeated three times, and the average values and standard deviation was were recorded.

### 3.5. Isolation of Secondary Metabolites

A sample of the methanolic extract was dechlorophyllated by open column chromatography on reverse phase silica RP-18 (40–63 µm) with methanol-:water 80:20 as eluent. A series of 20 mL portions were collected and then combined according to chromatographic similarity in eight fractions, of which fraction 2 was the most abundant. This fraction was further separated in open column chromatography using direct phase silica and ethyl acetate: methanol: water 90:4:1 as eluent. Fraction 4 was subject to further separation steps through Flash chromatography (Buchi Reveleris^®^ PREP, Flawil, Switzerland) using an RP-18 silica column, and a methanol: water elution gradient from 40:60 to 70:30. From fraction 13, four compounds were isolated by preparative thin layer chromatography (PTLC).

### 3.6. Characterization and Identification of Secondary Metabolites

Isolated secondary metabolites were identified through ^1^H and ^13^C NMR spectra in a Bruker 500/125 MHz (Billerica, MA, USA) spectrometer using deuterated solvents: methanol and acetone. The spectra were complemented by 2D experiments: Homonuclear Correlation Spectroscopy (COSY), Total Correlation Spectroscopy (TOCSY), Heteronuclear Multiple Bond Correlation (HMBC), and Heteronuclear Multiple Quantum Correlation (HMQC), to aid with the structural elucidation. The candidate compound identity was confirmed by comparison with published results [87,88,89,90].

## 4. Conclusions

The phytochemical composition of *T. volubilis* has been partially determined for the first time. The phytochemical screening of the methanolic extract of the aerial parts of the species shows the presence of alkaloids, terpenoids, tannins, and flavonoids, similar to the composition of polar extracts from other species of the genus.

The methanolic extract shows strong antioxidant activity, which can be partially attributed to the presence of phenolic compounds. Four bioactive flavonoid compounds, avicularin, quercitrin, afzelin, and amentoflavone, have been isolated from the extract. These compounds exhibit biological activity that supports the reported ethnopharmacological uses of the plant, both in vitro and in vivo, and can be associated with their antioxidant bioactivity.

More studies are needed to completely determine the phytochemical makeup of *T. volubilis*, and to establish its biological activity and potential therapeutic use to fully validate the existing ethnopharmacological claims and develop better, low-toxicity treatments.

## Figures and Tables

**Table 1:** Traditional uses of New World *Tragia* species.

Species	Region	Uses	Refs.
*Tragia cordata* Michx.	USA	Urinary tract conditions	[13]
*Tragia geraniifolia* Klotzsch ex Müll. Arg.	Bolivia, Paraguay, Uruguay, Argentina	Emollient	[14]
*Tragia nepetifolia* Cav.	USA, Mexico	Snakebite	[11]
*Tragia pinnata* (Poir.) A. Juss.	Brazil, Argentina	Emollient	[15]
*Tragia ramosa* Torr.	USA, Mexico	Ant bite	[16,17]
*Tragia uberabana* Müll. Arg.	Brazil	NS	[18]
*Tragia volubilis* L.	Mexico to Argentina	See below	[10]
*Tragia yucatanensis* Millsp.	Mexico, Belize, Honduras	Burns, rheumatism	[19]

**Table 2:** Ethnomedical uses of *Tragia volubilis* L.

Use	Plant Organ	Country	Preparation/Administration	Refs.
Analgesic	Stem, leaves	Cameroon	Decoction	[31]
Antirheumatic	Leaves, branches	Colombia	Lightly whip affected joints	[32]
Anti-ulcer	NS	Brazil	NS	[33]
Blood pressure	Leaf	Brazil	Infusion; oral	[34]
Cancer prevention	Leaf	DutchCaribbean	Infusion, oral	[35]
Diuretic	NS	Argentina	NS	[36]
Fertility	Stem, leaves	Cameroon	Decoction	[31]
Skin ulcers	Aerial parts	Cuba	Plant juice mixed with salt, topical	[37]
Sudorific	Root	Cuba	Decoction, oral	[37,38]
Venereal diseases	Leaves	Mexico	Decoction, NS	[30]
Venereal diseases	Root	Cuba	Decoction, NS	[37]
Wound anti-infective	Branches with leaves	Colombia	NS, oral	[39]
Wound anti-infective	Branches with leaves	Colombia	Decoction, topical	[32]

NS: not specified.

**Table 3:** Phytochemical screening of *T. volubilis* methanolic extract.

Compound Family	Presence	Test
Protein	−	Biuret
Carbohydrates	++	Fehling
Fats	−	Sudan
Alkaloids	+++	Dragendorff
Terpenoids	+	Lieberman Burchard
Flavonoids	++	Shinoda
Saponins	−	Foam
Quinones	−	Bornträger
Tannins	+++	Ferric Chloride Assay

−: Absence. +: Small presence. ++: Medium presence. +++: Strong presence.

**Table 5:** Antioxidant activity of compounds **1**–**4**.

Compound	DPPH IC_50_ (µM)	Refs.
**1**	71.68 ± 0.06	[60]
**2**	68.26 ± 1.37	[60]
**3**	14.89 ± 1.71	[61]
**4**	10.64 ± 0.15	[62]

**Table 6:** Selected biological activities of isolated compounds from *T. volubilis*.

Compound	Activity	Biological Model	Effect	Refs.
Avicularin (**1**)	Anti-fungal	*Candida albicans*.	MIC: 4 μg/mL	[66]
	Antiproliferative	SCC13 cells	Dose and time-dependent apoptosis induction	[67]
	Antirheumatic	Human synovial Rheumatoid arthritis cells	Dose-dependent viability inhibition and apoptosis induction	[68]
Quercitrin (**2**)	Antidiabetic	Male albino Wistar rats, streptomycin-induced diabetes	Glucose homeostasis improvement (*p* < 0.05) effect at 30 mg/kg dose.	[69]
	Anti-ulcer	Female Swiss mice	1.38 mg/kg reduces MPO activity	[70]
Afzelin (**3**)	Antibacterial	*Pseudomonas aeruginosa*	MIC: 31 µg/mL	[71]
	Diuretic	Female Wistar rats	Calcium-sparing diuretic activity. Nephroprotective	[72]
	Anti-ulcer	Female Swiss mice	0.078 mg/kg reduces MPO activity	[70]
Amentoflavone (**4**)	Cytotoxic	HeLa cells	IC_50_ 20.7 μM	[73]
	Antirheumatic	Osteoarthritis-induced Wistar rats	Improvements in incapacitation, motor activity, allodynia, and hyperalgesia parameters	[74]

Notes: MPO: Myeloperoxidase; MIC: minimum inhibitory concentration; IC_50_: 50% inhibitory concentration.

## Data Availability

All relevant data is already in the article.
